# Predictors for Perioperative Blood Transfusion in Patients Undergoing Open Cystectomy and Urinary Diversion and Development of a Nomogram: An Observational Cohort Study

**DOI:** 10.3390/jcm10132797

**Published:** 2021-06-25

**Authors:** Dominique Engel, Christian M. Beilstein, Pascal Jerney, Marc A. Furrer, Fiona C. Burkhard, Lukas M. Löffel, Patrick Y. Wuethrich

**Affiliations:** 1 Department of Anaesthesiology and Pain Medicine, Inselspital, Bern University Hospital, University of Bern, CH 3010 Bern, Switzerland; dominique.engel@insel.ch (D.E.); christian.beilstein@insel.ch (C.M.B.); doc@jerney.ch (P.J.); lukas.loeffel@insel.ch (L.M.L.); 2 Department of Urology, Inselspital, Bern University Hospital, University of Bern, CH 3010 Bern, Switzerland; marcalain.furrer@insel.ch (M.A.F.); fiona.burkhard@insel.ch (F.C.B.); 3 Department of Urology, Royal Melbourne Hospital, The University of Melbourne, Melbourne, VIC 3050, Australia

**Keywords:** blood transfusion, predictors, cystectomy

## Abstract

Open radical cystectomy is associated with a substantial rate of perioperative blood transfusion. Early detection of potentially modifiable perioperative factors could reduce the need for perioperative blood transfusion and thus positively impact the outcome. We conducted an observational, single-center cohort study of 1168 patients undergoing cystectomy. Perioperative blood transfusion was defined as the need for packed red blood cells and/or fresh frozen plasma units within the first 24 h after the initiation of surgery. Multiple logistic regression analysis was performed to model the association between risk factors and blood transfusion, and a nomogram was developed. Blood transfusion occurred in 370/1168 patients (31.7%). Significant predictors were age (OR: 1.678, (95% CI: 1.379–2.042); *p* < 0.001), blood loss ratio (6.572, (4.878–8.853); *p* < 0.001), preoperative hemoglobin (0.316, (0.255–0.391); *p* < 0.001), tumor stage (2.067, (1.317–3.244); *p* = 0.002), use of oral anticoagulants (2.70, (1.163–6.270), *p* = 0.021), and interaction between female sex and blood loss ratio (1.344, (1.011–1.787); *p* = 0.042). Of the major predictors found to affect perioperative blood transfusion, two can be influenced: blood loss ratio by meticulous surgery and hemoglobin by preoperative optimization. Others such as age or advanced disease are not modifiable. This emphasizes the importance of optimal management of patients prior to surgery.

## 1. Introduction

Radical cystectomy, pelvic lymph node dissection, and urinary diversion remain the standard of care for patients with muscle-invasive bladder cancer [1]. Despite substantial improvement over the last decades in terms of surgical technique including robotic-assisted surgery, this procedure is still associated with substantial intraoperative blood loss and transfusion of packed red blood cells (PRBC) with or without administration of fresh frozen plasma (FFP) [2,3]. While robotic-assisted radical cystectomy has been associated with reduced blood loss and blood transfusion and is gaining more relevance, open radical cystectomy is still widely performed worldwide [4]. An overly restrictive blood transfusion strategy has been shown to increase the risk of the composite event “inadequate oxygen supply plus mortality” in a systematic review and meta-analysis, including context-specific conditions like patients’ characteristics and clinical setting [5]. On the other hand, the reported harm of blood transfusions is manifold. Perioperative blood transfusions have been associated with worse outcomes following cystectomy [6,7,8,9,10,11,12]. Blood transfusions have an immunomodulatory and immunosuppressive effect in the perioperative period [13,14], which predisposes patients to an increased rate of infection [15,16]. The intraoperative period is also crucial as the physiological response to surgery can induce a pro-tumor environment and thus negatively impact the control of minimal residual disease or metastatic spread of tumor cells. In this setting, the administration of PRBC and FFP with its profound negative effects on the immune system is detrimental [17]. In addition, blood transfusions per se are associated with adverse reactions like transfusion-related acute lung injury, which is considered the leading cause of blood transfusion-related morbidity. Finally, blood transfusions result in a relevant increase in overall medical costs [18,19].

Cystectomy patients are particularly inclined to receive blood transfusions as a substantial number of patients (up to 50%) are anemic preoperatively [20,21]. Based on these findings, we aimed to determine the risk factors for early blood transfusions, to identify potentially modifiable pre- and intraoperative factors, and to develop a nomogram to identify patients in need of preoperative optimization to reduce perioperative blood transfusions in patients planned for cystectomy.

## 2. Materials and Methods

This study reports a single, tertiary, high case-load center cohort study in accordance with the STROBE recommendations. Ethical approval was provided by the Ethics Committee of Canton Bern, Switzerland (2 June 2016, KEKBE 2016-00660), and the need for informed consent was waived.

### 2.1. Study Population and Data Collection

We identified 1174 consecutive patients at the Department of Urology, Bern University Hospital, between 4 January 2000 and 13 June 2016. We excluded six (0.51%) patients with insufficient follow-up or incomplete data, leaving 1168 for the final analysis.

All patient data evaluated were from a prospectively maintained database, including all relevant preoperative and oncological variables. Surgical factors were duration of surgery, intraoperative blood loss, previous surgery, perioperative need for PRBC and FFP transfusion, defined as blood transfusion within the first 24 h after the beginning of surgery [22,23].

Our institution has performed a similar and standardized open surgical technique for cystectomy and urinary diversion for the last 20 years, which has been described previously according to the Studer’s technique [24,25]. All patients were followed prospectively. During the observed period, PRBC was administered if hemoglobin values decreased to <80 g/L (or <100 g/L in patients with coronary artery disease). FFP was administered in the case of clinical coagulopathy or secondary to coagulation factor deficiency.

### 2.2. Outcome Measures

The need for a blood transfusion cannot be based on absolute blood loss alone but also depends on the patient’s blood volume. We, therefore, decided to implement a blood loss ratio based on the following calculations [26]:

Indexed blood volume: $BVi=\frac{70}{\sqrt{\left(\frac{BMI}{22}\right)}}$
Estimated blood volume: BVe=BVi∗Weight
Blood loss ratio: $BLr=\frac{BVe}{BLa}$

where BVi = indexed blood volume, BMI = body mass index, BVe = estimated blood volume, BLr = blood loss ratio, BLa = absolute blood loss

### 2.3. Statistical Analysis

Baseline, intraoperative, and postoperative variables between patients who received blood transfusions and patients who did not were compared. Continuous variables are presented as median and interquartile ranges (IQR) and categorical variables as frequencies (%). Group comparisons were calculated using the χ^2^ test for categorical and the Wilcoxon test for continuous variables. To identify independent risk factors for blood transfusions, we performed multiple logistic regression analyses. We first partitioned data into a training (90%) and a testing set (10%) to avoid overfitting and to assess model performance (ROC-AUC) on previously unseen data. We performed a stepwise selection procedure minimizing the Akaike information criterion (AIC) using the MASS package for R for the variable section. BMI, Charlson comorbidity index (CCI), use of blood thinners, previous surgery, tumor stage (using polynomial contrasts), positive nodal stage and neoadjuvant chemotherapy, duration of surgery, use of norepinephrine, and the total amount of fluid administered as well as the presence of and all two-way interactions between blood loss ratio, preoperative hemoglobin, age, and gender were evaluated for predictors of transfusion.

**Figure A1:**
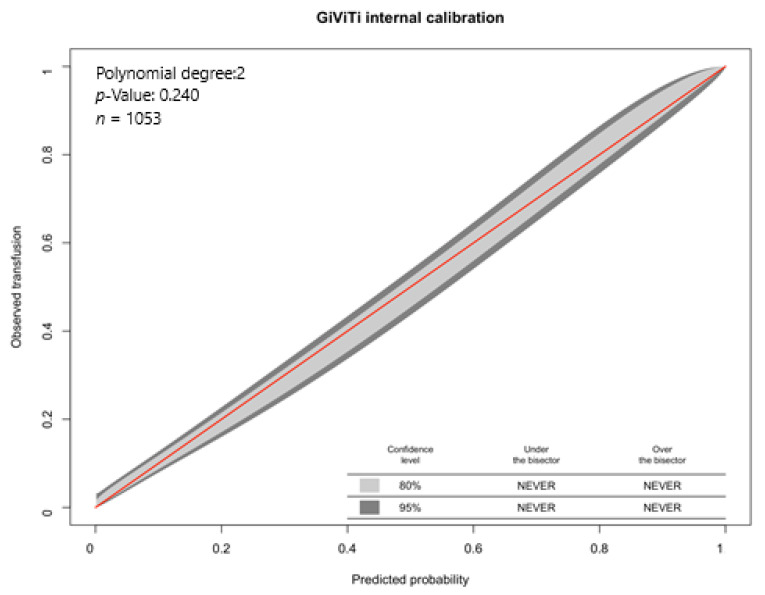
Gruppo Italiano per la valutazione degli interventi in Terapia Intensiva (GiViTi) internal calibration.

**Figure A2:**
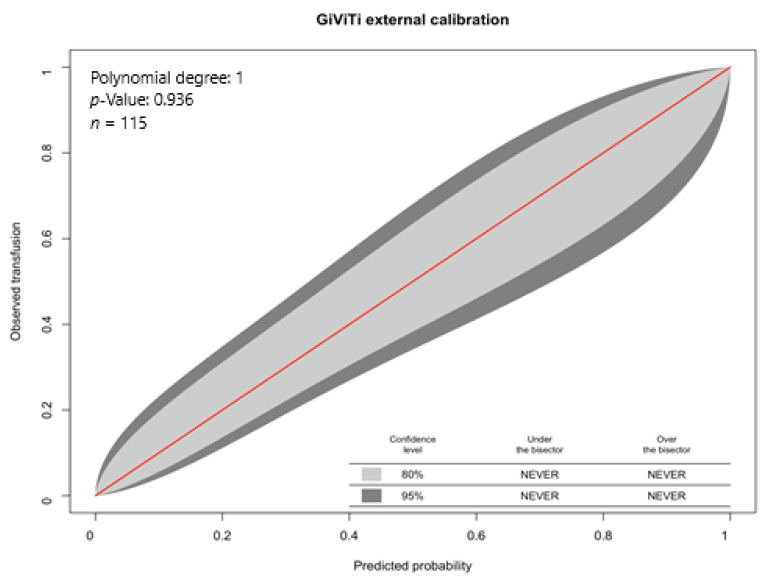
GiViTi external calibration.

All continuous variables were normalized. Multicollinearity between predictors was checked using the variance inflation factor. Marginal means were calculated for ordinal categorical predictors using the “emmeans” package with multiplicity adjustment using the multivariate t distribution method. Model fit was assessed with the le Cessie–van Houwelingen–Copas–Hosmer global goodness of fit test, the Hosmer–Lemeshow test, binned residual plots (“performance” package), and GiViTi calibration plots (“givitiR” package) [27,28] (Appendix A, Table A1, Figure A1 and Figure A2).

A nomogram was used to visualize the final logistic regression model using the “rms” package [14]. We chose the inverse-logit transformation of the linear predictor to depict the probabilities of the outcomes.

**Figure A3:**
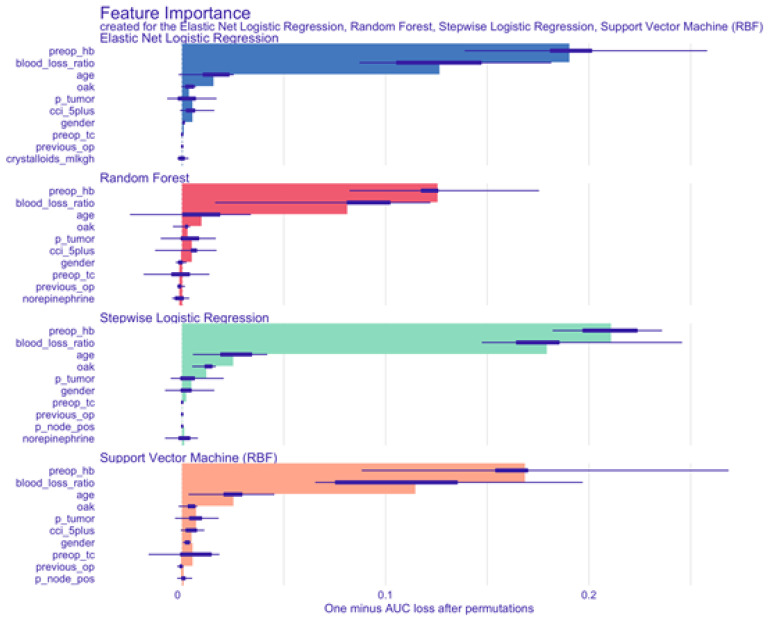
Feature importance plot with bars showing the loss in ROC-AUC when the corresponding term is removed.

We conducted a sensitivity analysis using modern machine learning models, especially elastic net logistic regression, random forest classification, and support vector machine. The results are depicted in the Appendix B and Appendix C (Table A3 and Figure A3).

A two-sided *p* < 0.05 was considered significant. Analyses were performed using the R software environment (R Foundation for Statistical Computing, Vienna, Austria, Version 4.0.2).

The sample size was based on a consecutive series of patients who underwent cystectomy and urinary diversion during the observation time. No formal statistical power calculation was calculated before the study.

## 3. Results

Overall, 370/1168 patients (31.7%) received blood transfusions within 24 h after the beginning of surgery. In the univariate analysis, patients in the transfused group were older (71 (64–77) vs. 67 years (59–74); *p* < 0.001), more often had neoadjuvant chemotherapy (20.3% vs. 12.7%; *p* = 0.001), were more often taking oral anticoagulants (5.7% vs. 2.9%; *p* = 0.03), had lower preoperative hemoglobin values (120 (104–131) vs. 135 g/L (123–145), *p* < 0.001) and were more comorbid (*p* < 0.001). There was a higher proportion of women (37.8% vs. 29.9%, *p* = 0.009) and more with advanced disease (positive nodal stage (*p* = 0.009) and tumor stage (*p* < 0.001)) in the transfused group. Duration of surgery was longer in transfused patients (409 (351–454) vs. 390 min (345–426); *p* < 0.001), and blood loss was higher (1400 mL (1000–2088) vs. 900 mL (700–1245); *p* < 0.001). This last observation persisted when blood loss ratio was applied (0.31 (0.22–0.42) vs. 0.19 (0.14–0.26). Consequently, the amount of crystalloids administered was higher 5.3 mL/kg/h (3.80–7.06) vs. 4.5 mL/kg/h (3.30–6.02); *p* < 0.001) in transfused patients (Table 1).

**Figure 1:**
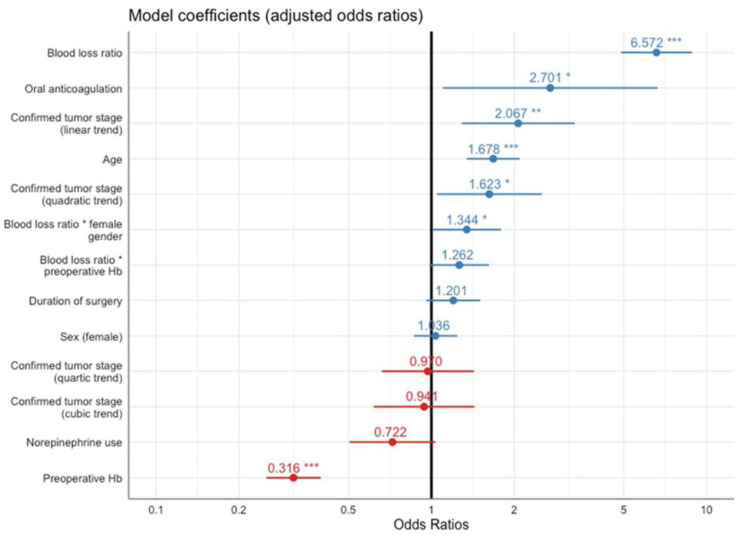
Effect plots for the predictors included in the final model (multiple logistic regression with adjusted odds ratio). Asterisks show the level of significance, *** for *p* < 0.001, ** for *p* < 0.01, * for *p* < 0.05.

The multiple logistic regression model is shown in Figure 1. ROC-AUC assessed on an independent test set was 0.87. The model showed a well-calibrated goodness of fit (Hosmer and Lemeshow C test: *p* = 0.617, Hosmer and Lemeshow H test: *p* = 0.100 and le Cessie–van Houwelingen–Copas–Hosmer test: *p* = 0.525). Diagnostic performance indicators were 65% sensitivity and 91% specificity. Multiple logistic regression detected blood loss ratio (OR: 6.572 (95% CI: 4.878–8.853); *p* < 0.001), oral anticoagulants (2.701 (1.163–6.270), *p* = 0.021), preoperative hemoglobin (0.316 (0.255–0.391), *p* < 0.001), age (1.678 (1.379–2.042), *p* < 0.001), and tumor stage (linear trend: 2.067 (1.317–3.244), *p* = 0.002 and quadratic trend: 1.623 (1.059–2.489), *p* = 0.026) as significant predictors for blood transfusions (Table 2). The interaction between female sex and blood loss ratio was significant (1.344 (1.011–1.787), *p* = 0.042). Table 3 shows the estimated marginal means for confirmed tumor stage with implemented polynomial contrasts. With more advanced tumor stages, there was a significantly increased risk for blood transfusions: pT3-4 vs. pT0-2 (OR: 2.028 (95% CI: 1.251–3.287), *p* = 0.001).

**Figure 2:**
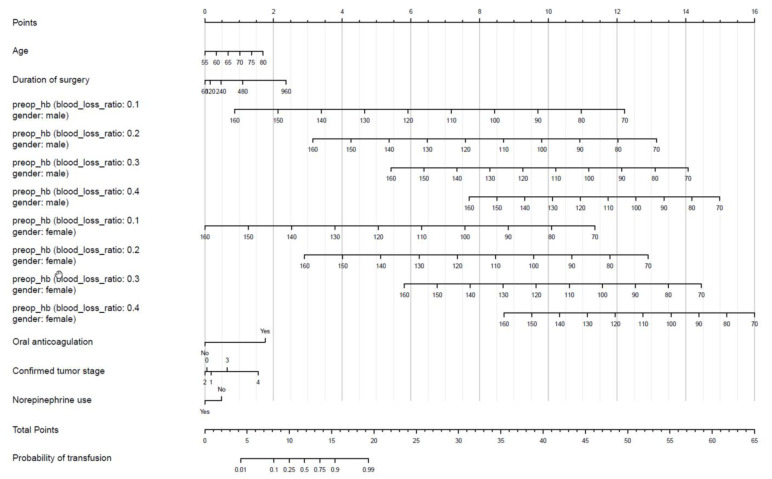
Nomogram of the logistic regression model to predict early perioperative blood transfusion.

**Figure A4:**
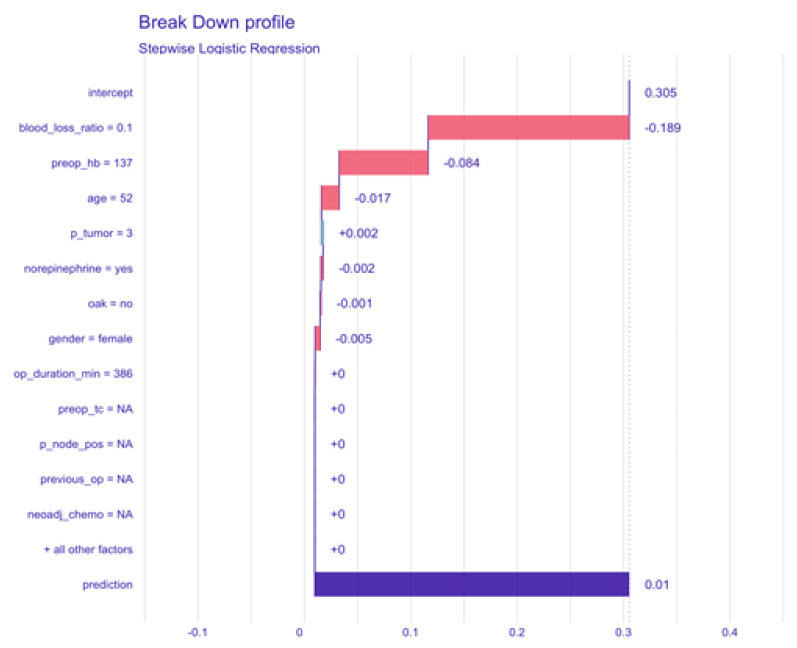
Younger female patient with normal hemoglobin level and lower blood loss (probability of transfusion: p_trans_ = 1%).

**Figure A5:**
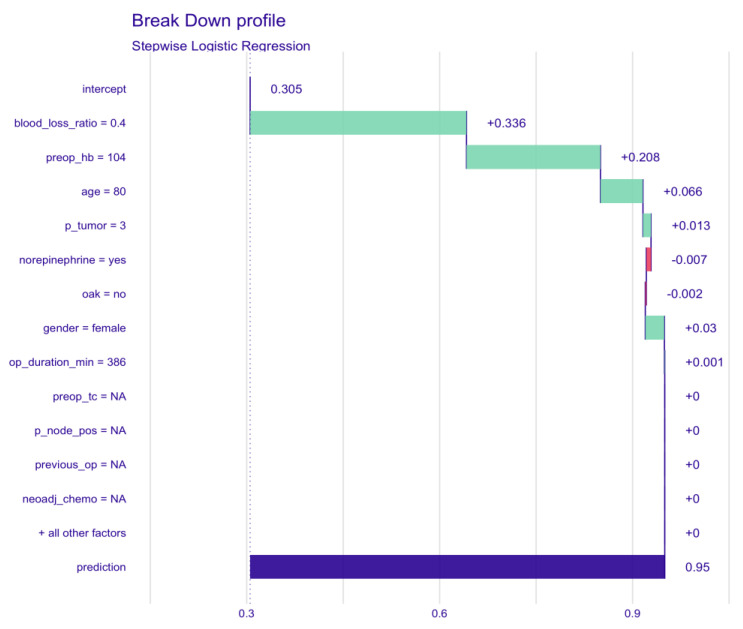
Older female patient with lower hemoglobin levels and higher blood loss (p_trans_ = 95%).

**Figure A6:**
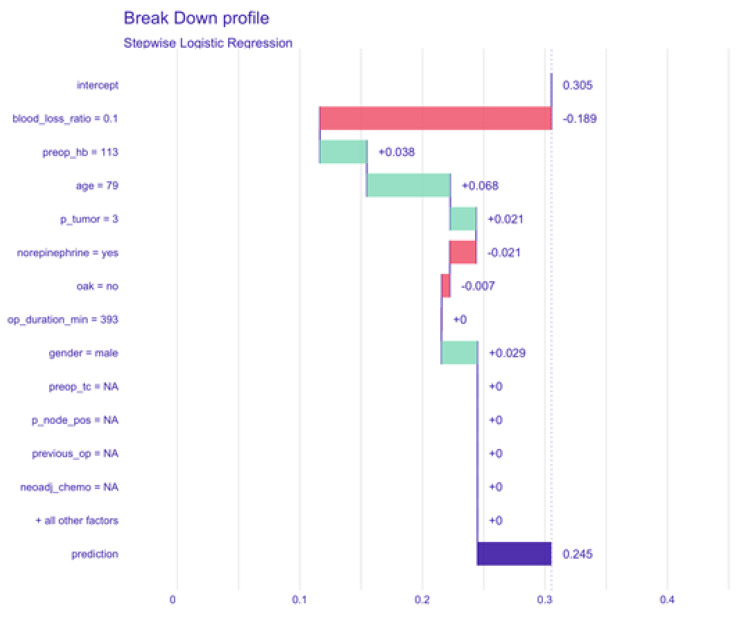
Older male patient with lower hemoglobin levels and lower blood loss (p_trans_ = 24.5%).

**Figure A7:**
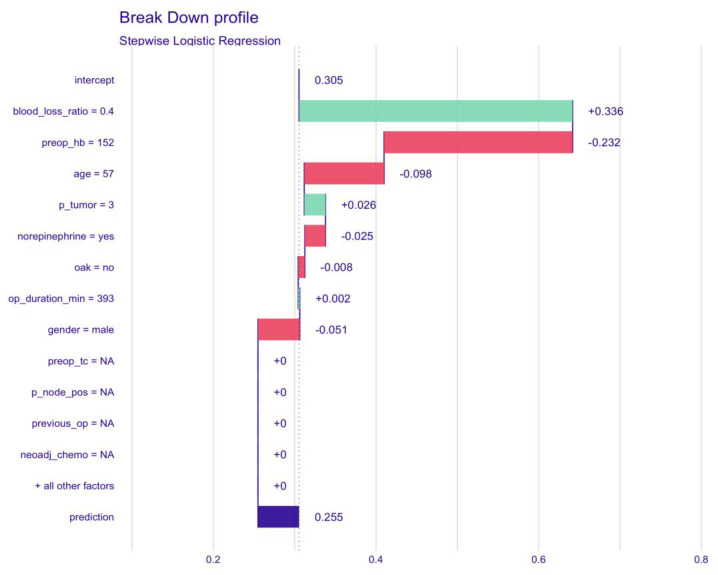
Younger male patient with higher hemoglobin levels and higher blood loss (p_trans_ = 25.5%).

**Figure A8:**
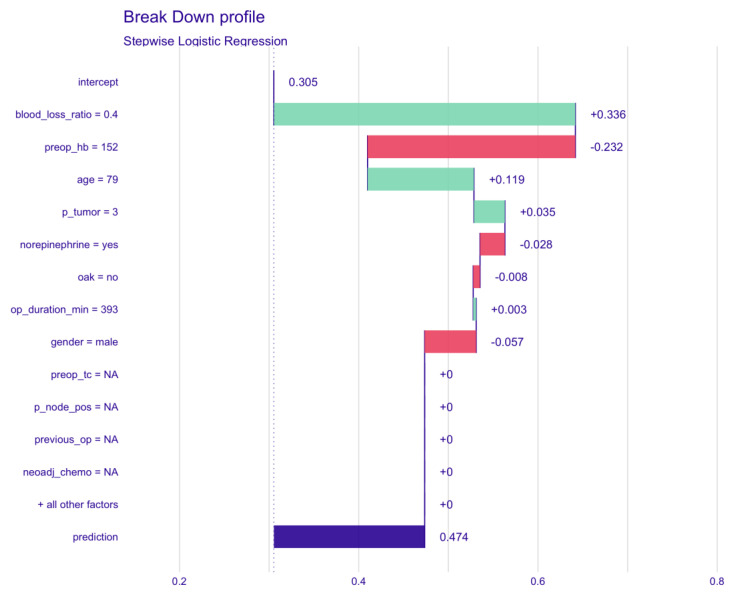
Older male patient with higher hemoglobin levels and higher blood loss (p_trans_ = 47.4%).

Figure 2 shows the nomogram for predicting blood transfusions, based on the final multiple logistic regression model. Exemplary patients are presented in Appendix D (Figure A4, Figure A5, Figure A6, Figure A7 and Figure A8) and Appendix E (Table A4, Table A5, Table A6, Table A7, Table A8 and Table A9).

The sensitivity analyses using sophisticated machine learning models showed a non-inferiority of our traditional logistic regression model. We thus favored it because of the better interpretability (Appendix B and Appendix C).

## 4. Discussion

This study aimed to detect predictors for perioperative blood transfusion and to develop a nomogram to predict the need for transfusion in patients undergoing cystectomy. In our cohort, we found an incidence of blood transfusion of around 30%, comparable with the current literature [29,30].

Blood loss ratio, preoperative hemoglobin, and advanced age had the strongest association with the likelihood of receiving a blood transfusion. Other relevant factors were the use of oral anticoagulants, advanced tumor stage, and interaction of female sex with blood loss ratio.

Age and tumor stage are given for the perioperative period, but their impact on the probability of a blood transfusion is limited as they contribute to the total score in the nomogram by less than 2 points. This holds true for the use of oral anticoagulants as well.

However, we identified two potentially modifiable factors that highly influence the probability of blood transfusion. These are easily discernable in the developed nomogram by a thick line indicating their predominance: preoperative hemoglobin and blood loss ratio outweigh all other factors by far, as they contribute up to 16 points to the total score in the nomogram.

The leading and most evident protective factor is a high circulating oxygen transport capacity, thus a high preoperative hemoglobin value. Its higher impact compared to other factors like duration of surgery, tumor stage, or oral anticoagulation is striking, particularly for female patients. This further emphasizes the importance of a solid preparation of patients by implementing standardized patient blood management concepts prior to cystectomy. Preoperative anemia should be corrected accordingly, through intravenous administration of an iron formulation, which results in a more rapid response compared to the oral formulation [31,32]. In addition, vitamin B12 and folic acid are also recommended for intravenous iron administration [33].

Furthermore, the use of continuous low-dose norepinephrine intraoperatively was included in the final model and nomogram, albeit not reaching statistical significance. This has been shown previously and is now confirmed in a large series of more than 1000 patients [34,35]. The protective effect of norepinephrine against blood transfusion is most likely attributed to vasoconstriction induced by the pre-emptive/concomitant use of a predominantly α-adrenergic vasopressor with only mild but dose-dependent β-adrenergic effects [29,35].

Female sex has been associated with an increased rate of blood transfusion in cardiac and major non-cardiac surgery [36,37,38]. Furthermore, Siegrist et al. found in a large cystectomy cohort that blood loss and transfusion rate were higher in 280 females [39]. In our series, this is only true if interacted with the blood loss ratio, potentially due to the lower circulating blood volume in females. Based on our nomogram, anemic female patients have a nearly twofold higher risk of receiving a blood transfusion than male patients with a similar blood loss ratio. In addition, we confirmed once again that the type of urinary diversion has no impact on blood loss and the rate of blood transfusion [34].

Although blood transfusions can be lifesaving and necessary in some cases, and they are safer than they have ever been, they still negatively influence the immune system. Blood transfusions are associated with promoting a proangiogenic environment inducing tumor growth by releasing cytokines IL6, IL10, and TNFα, and regulatory T-cell activation suppressing the anti-tumoral Th1 response [14]. Notably, not only PRBC but also FFP induce a pro-inflammatory burden and have the potential to affect natural killer cell activity. FFP contains Th2 cytokines, which induce a dose-dependent release of TNFα and IL10 [40]. This cytokine response is known to be pro-tumoral.

The ideal perioperative blood transfusion strategy in major oncological surgery remains unclear. This is especially important for older comorbid patients because a too restrictive blood transfusion regimen could increase the risk of early major postoperative complications (ischemic, cardiogenic). On the other hand, a too liberal strategy raises the risk of thromboembolic events [5,41,42,43]. Thus, a careful balance between the short-term benefits of blood transfusions in terms of reduction in early major postoperative complications versus the long-term benefits in terms of better cancer-related outcomes should be made depending on the patient's characteristics. Meticulous surgical technique reducing blood loss, restrictive fluid administration avoiding hemodilution, normothermia reducing the risk of coagulopathy, and a restrictive threshold for transfusions of PRBC and FFP are valuable intraoperative options to reduce blood transfusion rates [29,44]. In the era of cryoprecipitate and fibrinogen concentrates, the indication for FFP should be reconsidered as it has been associated with transfusion-associated circulatory overload and transfusion-related acute lung injury. Malignancy and the disturbances in the perioperative period are known to induce prothrombotic stimuli, and administration of blood transfusions has been associated with an elevated risk of thromboembolic events [45]. Hence, the administration of PRBC and FFP units must be evaluated carefully.

The key finding of this study is that the most important and most efficient method to avoid blood transfusion is preoperative patient optimization as depicted in our nomogram. The cornerstone in preparing patients scheduled for cystectomy is to optimize hemoglobin levels in anemic patients to within the normal to high hemoglobin range (≥130 g/L in both sexes) as proposed in the international consensus statement on the perioperative management of anemia and iron deficiency [46]. Their recommendations include a perioperative care pathway to investigate and correct anemia. In order to minimize the risk of unfavorable transfusion-related outcomes, the implementation of patient blood management strategies is valuable, even if the time between decision and surgery is limited, to avoid postponing major cancer-related surgery [47].

## 5. Conclusions

The incidence of perioperative blood transfusions in patients undergoing cystectomy was around 30%. Of the predictors affecting blood transfusions, only blood loss ratio and preoperative hemoglobin can be addressed by meticulous surgery and surgical technique, or optimization of preoperative hemoglobin as depicted in our nomogram. Others, like age or advanced tumor disease, are not modifiable. This emphasizes the importance of optimal preparation of patients undergoing cystectomy and minimal bleeding during surgery. We, therefore, strongly suggest the implementation of solid patient blood management, as previously proposed, to increase preoperative hemoglobin concentration.

### Limitations

This is a retrospective study based on prospectively recorded data. We, therefore, cannot exclude deviation of the transfusion protocol due to clinical decision-making. This limits the generalizability of these findings. However, we were able to confirm, in part, precedent results. In addition, there is a high likelihood for a negative selection bias in the group receiving blood transfusions. Patients receiving blood transfusions were more likely to have more advanced oncological disease, were older, and more comorbid at the time of surgery. A randomized, controlled trial, which would be the ideal design to avoid bias and answer this question, seems ethically not justifiable.

## Figures and Tables

**Table 1:** Baseline, oncological, and surgical/anesthetic characteristics.

	Non-Transfused	Blood Transfusion	*p*-Value	SMD
**Baseline Characteristics**				
Number of Patients (%)	798 (68.3)	370 (31.7)		
Age (years) (median (IQR))	67.08 (59.21, 74.29)	71.05 (64.04, 77.41)	<0.001	0.296
Sex (female) (%)	239 (29.9)	140 (37.8)	0.009	0.167
BMI (kg/m^2^) (median (IQR))	25.56 (22.86, 28.72)	25.40 (22.73, 28.51)	0.710	0.039
Charlson Comorbidity Index (%)			<0.001	0.317
0	304 (38.1)	106 (28.6)		
1	131 (16.4)	48 (13.0)		
2	181 (22.7)	90 (24.3)		
3	98 (12.3)	56 (15.1)		
4	55 (6.9)	37 (10.0)		
5 and more	29 (3.6)	33 (8.9)		
Oral anticoagulation (%)	23 (2.9)	21 (5.7)	0.030	0.138
Thrombocyte aggregation inhibition (%)	87 (10.9)	40 (10.8)	1.000	0.003
Preoperative Hb (g/L) (median (IQR))	135.00 (123, 145)	120.00 (104, 130.75)	<0.001	0.826
Preoperative Tc (G/L) (median (IQR))	251.5 (211, 309)	267.00 (214.25, 338.75)	0.002	0.236
**Oncological characteristics**				
Confirmed tumor stage pT (%)			<0.001	0.414
0	201 (25.2)	59 (15.9)		
1	111 (13.9)	38 (10.3)		
2	193 (24.2)	75 (20.3)		
3	233 (29.2)	129 (34.9)		
4	60 (7.5)	69 (18.6)		
Confirmed positive nodal stage (%)	167 (20.9)	104 (28.1)	0.009	0.168
Neoadjuvant chemotherapy (%)	101 (12.7)	75 (20.3)	0.001	0.206
**Surgical/anesthetic characteristics**				
Previous surgery (%)	326 (40.9)	162 (43.8)	0.378	0.059
Duration of surgery (min) (median (IQR))	390 (345, 425.75)	408.50 (351, 454)	<0.001	0.293
Blood loss (ml) (median (IQR))	900 (700, 1245)	1400.00 (1000, 2087.5)	<0.001	0.782
Blood loss ratio (median (IQR))	0.19 (0.14, 0.26)	0.31 (0.22, 0.42)	<0.001	0.862
Norepinephrine use (%)	519 (65.0)	203 (54.9)	0.001	0.209
Crystalloids (ml/kg/h) (median (IQR))	4.5 (3.30, 6.02)	5.30 (3.80, 7.06)	<0.001	0.253

Abbreviations: Tc, thrombocytes; Hb, hemoglobin, SMD: standardized mean difference.

**Table 2:** Multivariate analysis of factors associated with the rate of early blood transfusion.

Variables	Odds Ratio	95% CI	*p*-Value
* Blood loss ratio	6.5717	4.8781–8.8532	<0.0001
* Oral anticoagulants	2.7007	1.1633–6.2699	0.0208
* Preoperative Hemoglobin (g/L)	0.3155	0.2545–0.3912	<0.0001
* Age (y)	1.6782	1.3794–2.0416	<0.0001
* Confirmed tumor stage (linear trend)	2.0670	1.317–3.2441	0.0016
* Confirmed tumor stage (quadratic trend)	1.6233	1.0587–2.4892	0.0263
* Confirmed tumor stage (cubic trend)	0.9408	0.6111–1.4484	0.7818
* Confirmed tumor stage (quartic trend)	0.9705	0.6541–1.4398	0.8816
* Duration of surgery < (min)	1.2011	0.9812–1.4701	0.0757
* Norepinephrine used	0.7220	0.5028–1.0367	0.0776
Sex (female)	1.0356	0.8666–1.2376	0.7002
Blood loss ratio * preoperative Hb	1.2619	0.9714–1.6393	0.0814
* Blood loss ratio * female gender	1.3443	1.0112–1.7871	0.0417

* = Variables included in the final model. Level of significance *p* < 0.05, CI = confidence interval, Hb = hemoglobin.

**Table 3:** Estimated marginal means for tumor stage (i.e., mean change contrasts for confirmed tumor stage).

Variable	Contrast	Odds Ratio	95% CI	*p*-Value
Confirmed tumor stage	0 vs. 1–4	1.2595	0721–2.199	0.6774
Confirmed tumor stage	0–1 vs. 2–4	1.6052	0.978–2.635	0.0662
Confirmed tumor stage	0-2 vs. 3–4	2.0278	1.252–3.285	0.0014
Confirmed tumor stage	0–3 vs. 4	2.3849	1.223–4.650	0.0057

Level of significance *p* < 0.05, CI = confidence interval.

## Data Availability

Data supporting generated results are available by the corresponding author.

## Appendix A

**Table A1:** Model diagnostics variance inflation factor (VIF) >10 indicating the presence of multicollinearity.

Variable	VIF	Df
Blood loss ratio	1.468	1
Oral anticoagulants	1.041	1
Preoperative hemoglobin	1.322	1
Age	1.180	1
Tumor stage	1.155	4
Duration of surgery	1.326	1
Norepinephrine	1.071	1
Female sex	1.091	1
Blood loss ratio x preoperative hemoglobin	1.109	1
Blood loss ratio x female	1.138	1

## Appendix B. Comparison with Machine Learning Models

All machine learning models were performed using the “tidymodels” package for R.

Elastic Net Logistic Regression

A 10-fold repeated 10-fold cross-validation was performed to find the best tuning parameters out of 50 combinations, which were chosen by the maximum entropy method. The best model was selected with a penalty of 0.0331 and a mixture parameter of 0.381 (0 being only ridge regression and 1 being only lasso regression), maximizing ROC-AUC.

Random Forest

A 10-fold repeated, 10-fold cross-validation was performed to find the best tuning parameters out of 100 combinations, which were chosen by the maximum entropy method. The best model was selected with mtry (number of variables available for splitting at each tree node) of 3 and a min_n (minimum number of data points in a node that are required for the node to be split further) of 34, maximizing ROC-AUC. The number of trees contained in the ensemble was always 1000.

Support Vector Machine

We used a radial basis function for the kernel. A 10-fold repeated, 10-fold cross-validation was performed to find the best tuning parameters out of 100 combinations, which were chosen by the maximum entropy method. The best model was selected with a cost of 3 and a sigma for the kernel of 0.0339, maximizing ROC-AUC.

Model performance

Model performance was assessed using a separate test set comprised of 10% of the whole data set as described in the Methods section of the main article.

**Table A2:** Comparison.

Model	Accuracy	ROC-AUC	Brier
Elastic Net Logistic Regression	0.783	0.867	0.137
Stepwise Logistic Regression	0.800	0.864	0.136
Support Vector Machine	0.800	0.864	0.133
Random Forest	0.800	0.829	0.147

## Appendix E. Nomogram Manual Score

**Table A3:** Age.

YEARS	POINTS
<65	0
65–79	1
>79	2

**Table A4:** Duration of surgery.

MINUTES	POINTS
<480	0
480–959	1
>960	2

**Table A5:** Preoperative hemoglobin—Blood loss ratio—Sex.

	BLOOD LOSS RATIO—MALE				BLOOD LOSS RATIO—FEMALE			
HB	0.1	0.2	0.3	0.4	0.1	0.2	0.3	0.4
160	1	3	5	8	0	3	6	9
150	2	4	6	8	1	4	7	10
140	3	5	7	9	3	5	8	10
130	5	6	8	10	4	6	9	11
120	6	8	9	11	5	7	10	12
110	7	9	10	12	6	8	11	13
100	8	10	11	13	8	10	12	14
90	10	11	12	13	9	11	13	14
80	11	12	13	14	10	12	13	15
70	12	13	14	15	11	13	14	16

**Table A6:** Oral anticoagulation.

	POINTS
NO	0
YES	2

**Table A7:** Confirmed tumor stage.

STAGE	POINTS
PT0	0
PT1	0
PT2	0
PT3	1
PT4	2

**Table A8:** Norepinephrine use.

	POINTS
NO	1
YES	0

**Table A9:** Probability of transfusion.

TOTAL POINTS	PROBABILITY OF TRANSFUSION
4	0.01
8	0.1
10	0.25
12	0.5
14	0.75
15	0.9
19	0.99
